# Vascular dysfunction in COVID-19 patients: update on SARS-CoV-2 infection of endothelial cells and the role of long non-coding RNAs

**DOI:** 10.1042/CS20220235

**Published:** 2022-11-11

**Authors:** Jaroslav Pelisek, Benedikt Reutersberg, Urs F Greber, Alexander Zimmermann

**Affiliations:** 1 Department of Vascular Surgery, University Hospital Zürich, Zürich, Switzerland; 2 Department of Molecular Life Sciences, University of Zürich, Switzerland

**Keywords:** cardiovascular disorder (CVD), COVID-19, endothelial cells (ECs), ong non-coding RNA, SARS-CoV-2, smooth muscle cells (SMCs)

## Abstract

Although COVID-19 is primarily a respiratory disease, it may affect also the cardiovascular system. COVID-19 patients with cardiovascular disorder (CVD) develop a more severe disease course with a significantly higher mortality rate than non-CVD patients. A common denominator of CVD is the dysfunction of endothelial cells (ECs), increased vascular permeability, endothelial-to-mesenchymal transition, coagulation, and inflammation. It has been assumed that clinical complications in COVID-19 patients suffering from CVD are caused by SARS-CoV-2 infection of ECs through the angiotensin-converting enzyme 2 (ACE2) receptor and the cellular transmembrane protease serine 2 (TMPRSS2) and the consequent dysfunction of the infected vascular cells. Meanwhile, other factors associated with SARS-CoV-2 entry into the host cells have been described, including disintegrin and metalloproteinase domain-containing protein 17 (ADAM17), the C-type lectin CD209L or heparan sulfate proteoglycans (HSPG). Here, we discuss the current data about the putative entry of SARS-CoV-2 into endothelial and smooth muscle cells. Furthermore, we highlight the potential role of long non-coding RNAs (lncRNAs) affecting vascular permeability in CVD, a process that might exacerbate disease in COVID-19 patients.

## Introduction

Coronaviruses (CoVs) belong to a large and heterogeneous subfamily of Coronaviridae, causing mostly respiratory disease and severe acute respiratory syndrome (SARS) which can be fatal without proper treatment [1–3]. In December 2019, a novel coronavirus SARS-CoV-2 was identified, causing a worldwide coronavirus disease 2019 (COVID-19) with over 6 million deaths [4–6]. COVID-19 may lead to the death of susceptible people. Notably, people with advanced age and comorbidities are at higher risk of severe complications. Comorbidities not only include chronic diseases like cancer, kidney and liver disease, asthma, COPD, diabetes, and heart failure but also smoking immunosuppression and drug abuse (https://www.cdc.gov/coronavirus/2019-ncov/need-extra-precautions/people-with-medical-conditions.html) [2,5,7–15].

Although COVID-19 is primarily a respiratory disease, other nonpulmonary manifestations have also been described, such as renal complications, neurological, gastrointestinal, and various cardiovascular disorders [9,10,16–26]. Particularly, patients suffering from cardiovascular disorder (CVD) have the highest risk of severe COVID-19 complications, leading often to death [27]. Intriguingly, CVD patients infected by SARS-CoV-2 have shown more severe pathophysiological changes in the lung compared to individuals without CVD. Endothelial cells (ECs) at the interface between blood and the underlying vascular tissue have also been described to be involved in the COVID-19 pathologies [14,28–37], possibly leading to inflammation and endothelial dysfunction. Currently, the clinical complications in COVID-19 patients with CVD are thought to be due to SARS-CoV-2 infection and subsequent dysregulation of ECs [12,36,38,39].

However, the evidence that ECs are infected by SARS-CoV-2 has been shown only by electron microscopy [28,31,40–42]. Interestingly, studies using immunohistochemistry (IHC) or *in situ* hybridization did not find compelling evidence of ECs infection by SARS-CoV-2 [43–45]. Accordingly, it has been even reported that ECs are at least partially resistant to SARS-CoV-2 infection [42,46]. Meanwhile, other factors have been described that might contribute to the viral entry into the host cells, including, e.g., disintegrin and metalloproteinase domain-containing protein 17 (ADAM17), the C-type lectin CD209L, and heparan sulfate proteoglycans (HSPG) [47–55]. SARS-CoV-2 has been reported to bind also to RGD motif (Arg-Gly-Asp) of integrins and to vimentin [56,57]. These structural proteins might increase and maintain the binding affinity of the viral S protein to the cell surface, facilitating SARS-CoV-2 entry. Thus, the entry mechanisms of SARS-CoV-2, particularly into ECs, have yet to be elucidated.

Recently, increasing evidence has revealed an important role of non-coding RNAs (ncRNAs) not only in cardiovascular disorders [58–62] but also in viral infection by regulating the entry mechanism and the host antiviral response [63–67].

In this review article, we discuss current data on SARS-CoV-2 entry into endothelial and smooth muscle cells and their dysfunction following COVID-19 infection in the context of CVD. Furthermore, we discuss intracellular mechanisms of infection variability and the pivotal role of non-coding RNA in COVID-19 and CVD.

## COVID-19 epidemiology and cardiovascular system

COVID-19 was first identified in Wuhan, the capital of the Hubei province of China, in December 2019. It spread rapidly throughout the world within a couple of months [2]. In August 2022, 600 million cases have been reported, resulting in over 6 million deaths worldwide [Word Health Organization, COVID-19 Dashboard, https://covid19.who.int/]. Many infected individuals have developed severe syndromes, with a global lethality of 1–3%, most of them with various comorbidities [68]. Significantly higher risks have been shown particularly in older patients, with a 3.6% mortality in 60–69 years old, 8.0% in 70–79 years old, and 14.8% in individuals older than 80 years [2].

Although COVID-19 is primarily a respiratory disease, it affects adversely also the cardiovascular system. Patients with CVD have the highest risk of contracting severe COVID-19 and death [11,15,23,24,27,69]. Besides older age (>65 years), the specific risk factors include hypertension, obesity, diabetes mellitus, chronic pulmonary disease, various vascular disorders, and heart failure [11,12,16–27,80]. CVD patients infected by SARS-CoV-2 tend to have more severe lung pathology compared to individuals without CVD. They also show elevated levels of inflammatory markers, such as C-reactive protein (CRP), interleukin (IL)-6, serum amyloid A (SAA), D-dimer, and fibrinogen, among others [9,39,70, 71–75].

## Human coronaviruses and infection cycle

Coronaviruses are enveloped, single-stranded, positive (+)-sense RNA viruses. Seven CoVs have been considered contagious, including the most recent one SARS-CoV-2, closely related to SARS-CoV 2002/2003 and more distantly related to MERS-CoV [76–78]. The remaining four CoVs (HCoV-NL63, HCoV-229E, HCoV-OC43, and HKU1) are endemic in the human population and typically cause mild respiratory complications compared with highly pathogenic SARS-CoV, MERS-CoV, and SARS-CoV-2 [79,80]. The common human CoVs account for 10–30% of the cold cases in adults but in rare cases they can cause life-threatening disease in infants, elderly or immunocompromised individuals.

CoVs encode four structural proteins: spike (S), membrane (M), envelope (E), and nucleocapsid (N) proteins [2,81]. The S protein is essential for virus binding to the host cell, entry, and infection [2,77,78,81]. SARS-CoV-2 and SARS-CoV are similar in their pathology, both binding to angiotensin-converting enzyme 2 (ACE2) receptor but have distinct transmission efficacy [78,82]. After docking to ACE2, the virus is processed by cellular transmembrane protease serine (TMPRSS2), which cleaves ACE2 and activates the S protein, facilitating virus entry into the host cell [2,83]. The infection leads to a massive immune response, often referred to as a cytokine storm [83,84].

Upon entering the host cell, the virus particle uncoats and releases its positive-stranded RNA into the cytosol [6]. Genome replication takes place on cytoplasmic double-membrane vesicles (DMVs) and membranous structures with small open double-membrane spherules [78,85]. The replication depends upon the synthesis of a viral replicase-transcriptase complex, composed of viral proteins as well as several host proteins [86]. The replication of CoV is associated with the endoplasmic reticulum (ER) leading to ER stress and may trigger apoptosis [79,86]. Furthermore, some of the viral proteins repress innate immunity and arrest the cell-cycle progression [86]. During virus assembly, the N protein binds to the viral genomic RNA and interacts with other structural proteins (S, E, and M) to assemble SARS-CoV particles. Virion morphogenesis is completed upon budding into organelles of the secretory pathway, including ER, Golgi, and intermediate compartment [79].

## SARS-CoV-2 cell entry through ACE2 receptor

**Figure 1:**
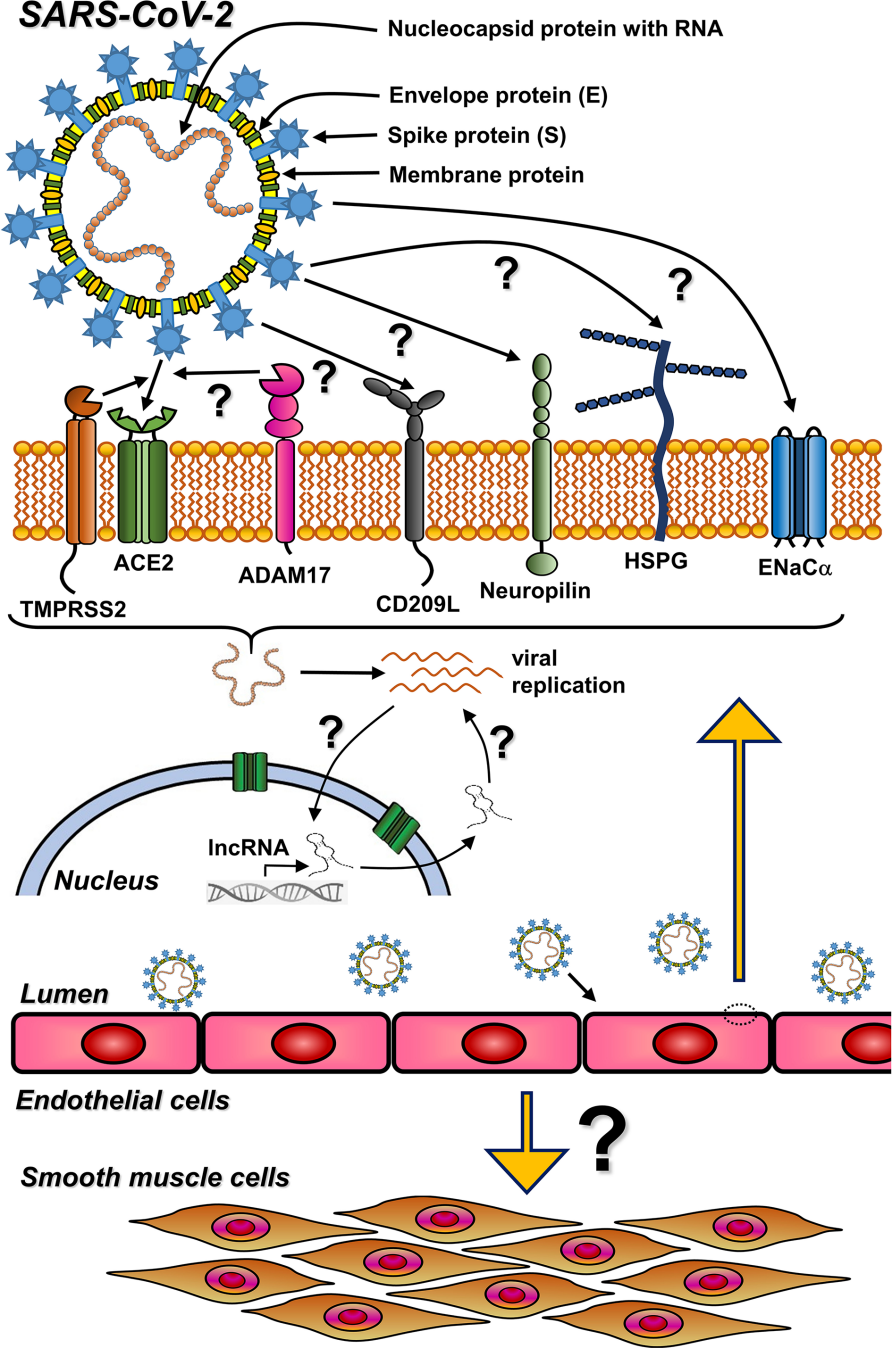
Potential entry mechanisms of SARS-CoV-2 in vascular cells and the role of lncRNA ACE2, angiotensin-converting enzyme 2; ADAM17, disintegrin and metalloproteinase domain-containing protein 17; CD209L, C-type lectin membrane protein; ENaC, epithelial sodium channel-α; HSPG, heparan sulfate proteoglycans; lncRNA, long non-coding RNA; TMPRSS2, transmembrane protease serine 2.

Early studies on CoVs have described high affinity of the receptor-binding domain (RBD) of the S protein to ACE2 receptor [2,87–90] (Figure 1). Thereby, SARS-CoV-2 has a higher binding affinity to ACE2 than SARS-CoV, which may increase infectivity [87,90]. As with many cell-associated viral receptors, the ectodomain of ACE2 can be shed from the cell surface by, e.g., metalloprotease ADAM17, and consequently circulates in blood and tissue fluids [91]. Soluble receptor ectodomain may limit SARS-CoV-2 invasion into the host cells by competing with the cell-associated ACE2 for SARS-CoV-2 binding [92,93]. Unlike SARS-CoV, SARS-CoV-2 is processed by the cellular transmembrane protease serine (TMPRSS2), which not only cleaves ACE2 but also activates the S protein, facilitating SARS-CoV-2 entry into the host cell [2,14]. Notably, ACE2 and TMPRSS2 are widely expressed in bronchial secretory as well as epithelial cells [94]. In absence of TMPRSS2, the virus and ACE2 undergo endocytosis, which together with protease-mediated shedding of ACE2 down-regulates the cell surface levels of ACE2 [68] and may ease the COVID-19 disease. In addition, ACE2 has also been considered a regulator of angiogenesis [37,95–97].

Although ACE2 and TMPRSS2 are the best-characterized CoV entry proteins, other cellular factors might be involved in virus entry and infection as well [50]. Furthermore, controversial results regarding SARS-CoV-2 infection of vascular cells exist [28,43,46].

## Alternative SARS-CoV-2 entry pathways

In general, CoVs can enter host cells by two mechanisms: cell membrane fusion, which engages ACE2 and TMPRSS2, or an endosomal pathway. Upon binding of spike (S) protein to ACE2 receptor the S protein is proteolytically primed by TMPRSS2 on the cell surface or by cathepsin B or L in the lysosomes [50,52,53] (Figure 1). Cathepsin B has been shown to be associated with SARS-CoV-2 infection of brain vascular cells, which do not express TMPRSS2 [51]. Cantuti-Castelvetri et al. and Daly et al. showed that the furin-cleaved S protein S1 binds to the neuropilin-1 receptors (NPR1) through the C-end rule peptide motive, which is present also in NRP2 and thereby significantly improved viral entry [47,98–100]. These results were extended by Giordo et al. who suggested neuropilins and CD209L, a C-type lectin membrane protein, highly expressed in human type II alveolar cells and lung ECs [47], as putative receptors facilitating SARS-CoV-2 entry into EC [100]. CD209L can mediate SARS-CoV-2 entry by interaction with the RBD domain of the viral S protein [101] (Figure 1). Other cell surface proteins and proteoglycans may also participate in SARS-CoV-2 entry. For instance, heparan sulfate proteoglycan (HSPG) was shown to be involved in the interaction between viral particles and infected cells [49,54]. Sialic acid was reported to bind to the spike proteins of SARS-CoV-2 and other human coronaviruses, possibly enhancing infection under favorable conditions [102,103].

Intriguingly, a molecular mimicry entry model of SARS-CoV-2 has also been suggested for human coronaviruses [48,89], where the S-protein may mimic the human α subunit of the epithelial sodium channel (ENaC-α). ENaC-α is associated with the renin–angiotensin system and regulates water homeostasis by modulating the level of sodium ions in lung airway tissue and controlling the volume of airway-surface liquids. Importantly, sodium channels require activation by furin cleavage to control the fluid reabsorption. Interestingly, the furin-like cleavage motif of S1/S2 proteins of SARS-CoV-2 is identical to that of ENaC-α (SARS-CoV-2 S1/S2 sequence: -RRAR x SVAS-, position 683-690; ENaC-α sequence: -RRAR x SVAS-, position 201-208; ‘x’ furin cleavage site) [102,104].

## ACE is a key regulator of the renin–angiotensin system

The renin–angiotensin system (RAS) plays a key role in the regulation of blood pressure, vascular tone, water and electrolyte homeostasis, cardiovascular and renal health [105–110]. Its activation is initiated by liver-derived angiotensinogen that is cleaved by renin into angiotensin I (Ang I). Ang I is further enzymatically modified into angiotensin II (Ang II) by ACE. The receptor ACE2, a homolog to ACE, cleaves Ang I and Ang II to Ang-(1-9) and Ang-(1-7), respectively [111]. Thereby, ACE promotes inflammation, vasoconstriction, and fibrosis, whereas ACE2 has anti-inflammatory, antiproliferative, vasodilatory, and antifibrotic effects [87].

The ACE2 receptor has various biological functions [88]. First, through Ang-(1-7) it exhibits various protective functions, including anti-inflammatory and anti-proliferative effects and reduction of oxidative stress, leading to vasodilation and maintenance of cardiovascular health [111]. Second, in the kidney and intestine, ACE2 activity is required for the uptake of amino acids across the epithelia [88]. Third, human CoVs use ACE2 as an entry portal into cells [88,93,112]. In addition, ACE2 has also been considered a regulator of angiogenesis [37,95–97,113]. Accordingly, increased levels of proangiogenic factors such as vascular endothelial growth factor (VEGF), fibroblast growth factor-2, and hypoxia-inducible factor-1α (HIF-1α) have been detected in COVID-19 patients [34,114–116]. Angiogenetic factors increase endothelial permeability, promoting the infiltration of inflammatory cells into the vessel wall. These circumstances contribute also to atherosclerosis in CVD patients [117–121].

All vascular cells, including ECs, SMCs, fibroblasts, and pericytes, express the ACE2 receptor as well [120,122]. Thereby, CVD can affect the expression of ACE2 promoting oxidative stress, inflammation and endothelial dysfunction [33]. In addition, cigarette smoking, diabetes, chronic pulmonary obstructive disease, certain tumors, age, and sex can also influence the expression of ACE2 [123].

## COVID-19 and endothelial cells in CVD

A common denominator of CVDs is endothelial dysfunction. ECs play a vital role in the maintenance of vascular haemostasis. Currently, the clinical complications in patients with COVID-19 and CVD are supposed to be the direct consequences of SARS-CoV-2 infection and dysregulation of ECs [13,14,31,32,38,116,124–128]. Under normal physiological conditions, the vascular endothelium regulates the systemic blood flow, immune response, coagulation, and tissue perfusion in line with the underlying SMCs or pericytes of the microvasculature [19,23,28,31,72,75,129–131].

Thereby, SARS-CoV-2 exacerbates or triggers endothelial damage either by infecting ECs directly or indirectly, by infecting other cell types, leading to hyperinflammation and impaired antiviral response [28,42]. Interestingly, similar processes lead to the development of CVD and promote atherosclerosis [23,37,106]. Thus, COVID-19-related hyperinflammation and endothelial damage might enhance and accelerate atherosclerotic progression, vulnerable plaques, and increase the risk of ischemic stroke or heart attack. Furthermore, an elevated neutrophil count in the blood of COVID-19 patients may enhance the formation of neutrophil extracellular traps (NETs) and thereby increase disease severity, thrombosis, and coagulopathy [116,132–134]. NETs are part of a rapid immune defence system against pathogens and initiate thrombosis by activating coagulation pathways [116,133]. In this context, increased levels of NET remnants have been detected in the serum of COVID-19 patients, including cell-free DNA, and factors promoting thrombosis and inflammation of the arterial wall [134].

Remarkably, endothelial pathology is also strongly associated with mitochondrial dysfunction and oxidative stress [33,135]. Many signaling pathways in inflammation are regulated by mitochondria and may have a role in COVID-19 pathology [33,136]. Mitochondrial reactive oxygen species (ROS) are potent oxidizers that can directly damage DNA, proteins, and lipids or activate downstream pathways that promote inflammation and endothelial dysfunction [13,137]. The infection of ECs by SARS-CoV-2 might contribute to mitochondrial dysfunction and enhanced oxidative stress, possibly initiating a feedback loop that promotes chronic inflammation and endothelial damage even after the virus has been cleared. Furthermore, oxidative stress and mitochondrial failure are associated with premature senescence of ECs, characterized by shortened telomeres, cell growth arrest, secretion of inflammatory cytokines, and growth factors [138]. Specifically, the NLRP3 inflammasome can be activated by ROS from dysfunctional mitochondria and is associated with EC senescence [139]. Cellular senescence in turn might have an impact on the regulation of ACE and the expression of ACE2, exhibiting a higher ACE/ACE2 ratio and thereby increasing susceptibility to SARS-CoV-2 [33]. Furthermore, COVID-19 infection can lead not only to mitochondrial dysfunction but may also facilitate the senescence of the host cell. These changes are irreversible, which is particularly relevant for patients recovered from COVID-19 disease [33]. Senescence promotes apoptosis and negatively affects nitric oxide synthase activity, thus increasing the risk of vascular damage and endothelial leakage [140]. The senescence-induced loss of nitric oxide would explain the results of Varga et al., who found patterns consistent with EC apoptosis in COVID-19 patients [31].

Notably, SARS-CoV-2 can induce endothelial-to-mesenchymal transition (EMT) in ECs characterized by the complete loss of endothelial features and acquisition of a fibroblast-like phenotype. This dedifferentiation process may involve TGF-β, a cytokine that is significantly increased in COVID-19 patients [33,141,142]. Consequently, a better mechanistic understanding of how SARS-CoV-2 affects ECs is necessary for the development of efficient therapeutic strategies for COVID-19 patients, particularly those suffering from CVD.

## SARS-CoV-2 infection of endothelial cells?

Viruses of many families can lead to endothelial infection and dysfunction, some of them involving cell adhesion molecules acting as receptors [28,35,124–126,130]. In turn, the activation of ECs may result in increased expression of adhesion molecules, such as ICAM-1, VCAM-1, E-selectin, P-selectin, MCP-1, and von Willebrand factor, thus enhancing the immune reaction and accumulation of inflammatory cells within the arterial wall [23,29,30,75,116].

Evidence that ECs are infected by SARS-CoV-2 comes from thin section analyses by electron microscopy [25,31,42,87]. For instance, Varga et al. found viral particles in ECs across vascular beds in different organs from COVID-19 patients [31]. Furthermore, other histological analyses of COVID-19 patients demonstrated that the increase in the inflammatory reaction is mainly associated with the dysfunctional endothelium, as well as with apoptotic bodies and cell death [22,42–44]. From these data, the authors suggested that SARS-CoV-2 can directly infect ECs. Notably, other studies found no evidence of viral particles in ECs using *in situ* hybridization or immunohistochemistry (IHC) on *ex vivo* tissue samples from patients with COVID-19 [28,43]. These results are not surprising given the sparsity of SARS-CoV-2 infected cells in the respiratory epithelium, which represents the entry gate for the virus into the body [116]. Further discrepancies have been observed between SARS-CoV-2 tropism and the cellular distribution of ACE2 and TMPRSS2 [43]. In addition, Bradley et al. found SARS-CoV-2-like particles post-mortem from COVID-19 patients only in renal ECs but not in other organs or arteries [143]. Another study using IHC *ex vivo* and in cell cultures did not detect any viral remnants in the vascular endothelium [45]. Yang et al. have reported that ECs derived from pluripotent stem cells are widely resistant to SARS-CoV-2 infection [46]. Furthermore, using *in situ* hybridization of liver biopsies, no viral particles were detected in the corresponding ECs [7]. It is thus not clear if thin-section electron microscopy in absence of immunostaining is able to unequivocally identify SARS-CoV-2-like particles [8,40,41,144]. High-resolution cryo-EM tomography might be required to identify the SARS-CoV-2 like structures. For example, Bernard et al. infected healthy human lung microvascular cells with SARS-CoV-2 *in vitro* and detected only modest viral RNA levels, indicating that ECs might be only moderately permissive to SARS-CoV-2 infection, even if they express both ACE2 and TMPRSS2 [28]. In other experiments, Hou et al. did not find any GFP-positive cells using the SARS-CoV-2-GFP reporter virus and human primary lung ECs [145]. Consequently, whether SARS-CoV-2 infects ECs has not yet been clarified and further studies are necessary.

Only a few studies focused on human ECs and a handful of them reported on the SARS-CoV-2 entry or expression of ACE2 [54,55,60,100,146–157]. In these studies, commercially available pulmonary, aortic, umbilical, or cardiac primary ECs were applied. So far, the results have been controversial, emphasizing the necessity of further research to clarify the impact of SARS-CoV-2 on ECs, particularly in CVD patients, in order to account for conditions of increased oxidative stress, inflammatory cytokines, reduced vasodilation and antithrombotic features, reflecting atherosclerotic changes within the arterial wall [13,135,158–160].

For instance, *in vitro* infections of HUVEC, microvascular endothelial cells (HLMVEC), coronary artery endothelial cells (HCAEC), and pulmonary arterial cells (HPAEC) with SARS-CoV-2 revealed no intracellular double-stranded viral RNA [150]. Furthermore, following SARS-CoV-2 infection, no intracellular double-stranded viral RNA was detected by immunofluorescence [161]. Using *in situ* hybridization, Targosz-Korecka et al. found no evidence for viral genomes in hepatic or endothelial cells [154]. The authors suggested that endothelial glycocalyx might shield the interaction of the SARS-CoV-2 S protein with the ACE2 receptor, thus protecting ECs from infection. This, however, contrasts the notion that SARS-CoV-2 efficiently infects highly polarized, well-differentiated human nasal and bronchial epithelial cells grown at the air–liquid interface even in presence of copious amounts of viscous mucus [162,163]. Potie at al. showed that blood plasma from COVID-19 patients facilitates glycocalyx shedding in ECs [155].

The evidence for the expression of ACE2, the main receptor for SARS-CoV2, on ECs is controversial [42]. Various studies and database searches have found only low expression of ACE2 receptor in human endothelial cells [25,42,87], even if ACE2 expression generally increases with age [164,165]. Nascimento Conde et al. found that healthy primary human ECs frequently lack the ACE2 receptor and resist SARS-CoV-2 infection. However, they become infected upon ectopic ACE2 expression indicating that either ECs are not primary targets of SARS-CoV-2 infection or ACE2-independent entry mechanisms are involved [146].

Regarding CVD, some studies suggested that ACE2 is significantly up-regulated in atherosclerotic ECs and in the elderly suggesting an aging-associated up-regulation of ACE2 [164–166]. Low-grade chronic inflammation has already been recognised as an important feature of aging, favoring the onset of various age-related diseases. These data might explain the higher risk and greater severity of COVID-19 disease observed in older individuals. Similar to the low-grade inflammation observed in the elderly, inflammatory reactions in COVID-19 pathogenesis, such as the up-regulation of IL1β and IFN-α, have been reported to stimulate ACE2 expression [104,105,107,123,164,165]. Taken together, further studies on COVID-19 both *in vivo* with CVD patients and *ex vivo* using vascular cells from different age groups are necessary to validate these hypotheses.

In summary, the global dysfunction of ECs together with the total or partial collapse of the endothelial barrier represents the critical step in the development of a severe course of COVID-19 disease contributing at the end to multiorgan failure and sepsis [116]. Due to the detachment and release of infected ECs into the circulation, SARS-CoV-2 infected ECs may disseminate and reach also other distant organs. Endothelial damage in turn leads to increased coagulation and other thrombotic events.

## COVID-19 and smooth muscle cells

Vascular SMCs are found in the tunica media and represent the most abundant cell type in the arterial wall [142]. They participate in vessel wall remodelling, provide mechanical strength and elasticity, regulate vascular tone and resistance, and connect vessels with the extracellular matrix [167,168]. SMCs play a pivotal role in CVD and atherosclerosis [169]. Phenotypic switching, apoptosis, necrosis, and the transdifferentiation of SMCs into macrophage-like or osteoblast-like cells significantly contribute to the disease progression through a weakening of the arterial wall [119]. SMCs are the most important cells synthesizing various components of the extracellular matrix to counteract their degradation by proteolytic enzymes activated in atherosclerosis.

As described above, RAS plays a key role in the regulation of vascular tone and in maladaptive vascular remodeling [105–110]. Consequently, RAS is tightly connected with the physiological function of SMCs [170,171]. It is generally accepted that the RAS has a dual function. On the one hand, there is an axis consisting of ACE-2/Ang (1-7)/MasR3 that exerts anti-inflammatory, antiproliferative, vasodilatory, and cardiovascular protective effects. The protective aspects of ACE2 are partly due to the down-regulation of various matrix metalloproteinases (MMPs) [170]. ACE2 deficiency in ApoE knockout mice deficient for ACE2 resulted in increased atherosclerosis and overexpression of MMP-2 and -9, destabilizing the vascular wall [172]. On the other hand, through the classical axis includingACE/Ang II/AT1 receptor (AT1R) and AT2 receptor (AT2R), it promotes inflammation, vasoconstriction, and fibrosis. Thereby, the Ang II effect is mediated through Ang II receptor type 1 (AT1) and 2 (AT2), leading to vasoconstriction and increasing cell proliferation, thus having favorable effects on the function of SMCs. The cross-talk between AT1R and AT2R has been described to have a significant effect on the cardiovascular system [170,171]. Consequently, the inhibition of the Ang II/AT1 signaling is an important pharmacological tool to prevent CVD related to vascular remodeling and might also improve the outcome of COVID-19 patients suffering from CVD. For instance, AT1 receptor blocker improves the effect of ACE2, thus increasing cardiovascular remodeling and normalizing fibrosis-associated signaling pathways in CVD [171]. The ACE2 inhibitors and AT1R blockers are considered the first-line treatment for patients with hypertension, suggesting their beneficial effects on the cardiovascular system under pathological conditions. Consequently, ACE2 treatment appears a promising target also for the management of patients with COVID-19, particularly those suffering from CVD. The activation of the Ang (1-7)/MAS receptor (MasR) axis may improve the physiological function of SMCs and vascular remodelling, being favourable in hypertensive patients infected by SARS-CoV-2 [171]. Savoia at al. suggest that independently of AT2R activation, MasR stimulation may contribute to the beneficial effects of ACE inhibitors or AT1R blockers on the pathological artery remodeling under hypertensive conditions.

Interestingly, RAS is also responsible for the regulation of ADAM17 expression. ADAM17-dependent ACE2 shedding is induced by Ang II via AT1R as well as by SARS-CoV-2 [173–175]. Following RAS activation, ADAM17 is up-regulated, leading to enhanced ectodomain shedding of ACE2 and increasing the soluble form of ACE2 in blood circulation [176]. The role of ACE2 shedding under physiological and pathological conditions is still insufficiently understood. Increased ADAM17 activity was detected in CVD, hypertension, diabetes mellitus, neurological, and oncological diseases [173,177].

In SMCs, ADAM17 is primarily responsible for the shedding of the epidermal growth factor receptor (EGFR) trans-activation induced by Ang II [178]. The ADAM17/EGFR signaling axis is one of the most key events by which RAS through Ang II affects vascular remodeling [179]. For instance, in AAA a significant up-regulation of ADAM17 was reported [180]. Deletion of ADAM17 in a mice model prevented AAA formation through attenuation of oxidative stress, inflammation, and extracellular matrix disruption [179]. Genome-wide transcriptional profiling identified AT1, ADAM17, and EGFR as important factors associated with vascular disorders induced by Ang II. Thus, ADAM17 represents potential therapeutic target for the prevention of CVD. In this context it is to mention that, despite low ACE2 expression, patients with CVDs have still higher COVID-19 mortality, caused probably by the imbalance between ADAM17 expression required for cleavage of ACE2 for virus uptake, and TMPRSS2, which is required for spike glycoprotein priming. Even if ACE inhibitors or AT1R blockers are well-established in the treatment of hypertension and other cardiovascular complications, their inhibition in patients with chronic comorbidities appears at the moment unjustified or at least questionable [152,177]. Further studies are necessary to evaluate the impact of ACE2 and AT1R inhibitors on the outcome of COVID-19 patients suffering from CVD. A critical aspect in the maintenance of the biological function of arteries is the cross-talk between ECs and SMCs. Aberrant EC–SMC communication is associated with various diseases, including atherosclerosis [169]. The cross-talk between SMCs and ECs is reciprocal. Alterations in SMCs may also change EC morphology and the expression of EC-specific genes [28,120]. Endothelial dysfunction is associated with changes in nitric oxide bioavailability and the release of various vasoactive compounds, conditions found when SARS-CoV-2 infects or affects ECs. This process may directly affect the function of SMCs and contribute to the severity of cardiovascular outcomes in COVID-19 patients. In this context, phosphodiesterase type 5 inhibitors (tadalafil) and estrogens increase the formation of endogenous nitric oxide and might thus exhibit a protective effect of these drugs in severe cases of COVID-19 [181].

Interestingly, despite the importance of SMCs in the maintenance of arterial function and the underlying pathologies, there are no data on the role of these vascular cells in COVID-19 patients suffering from CVD. Likewise, it is unknown whether SARS-CoV-2 can directly infect SMCs or whether and how far SMCs are affected by the dysfunctional ECs.

Only a handful of articles have explored the role of SMCs in the context of COVID-19 [148,182–187]. One of them focused exclusively on the expression of ACE2 in ECs, mentioning SMCs only on the side [183]. Marchiano et al. showed that human pluripotent stem cell-derived SMCs can be infected by SARS-CoV-2 [184]. Suzuki et al. treated pulmonary artery SMCs with the SARS-CoV-2 S protein [148], and the last study explored the effect of estrogen and testosterone on the expression level of ACE2 in human airway SMCs [183]. In the context of SMCs, it is again important to mention that there are significant differences in the expression levels of ACE2 between healthy and atherosclerosis-derived vascular cells [182]. Taken together, little is known about the role of SMCs in COVID-19 disease, particularly in patients suffering from CVD. Further studies are necessary, using e.g. CVD patient-derived vascular cells.

In this context, it is to note that SMCs in patients with CVD may be directly exposed to SARS-CoV-2. In advanced atherosclerotic lesions or aneurysms, the endothelial layer is largely damaged, enabling access of SARS-CoV-2 into the vessel wall to infect also the underlying SMCs, which express ACE2 [122,149]. Furthermore, the down-regulation of ACE2 in SARS-CoV-2-infected ECs leads to endothelial leakage and increased permeability, thus potentially allowing viral particles to access SMCs through the vessel wall [126,188]. Suzuki et al. [148] treated pulmonary artery SMCs with a recombinant SARS-CoV-2 spike protein S1 causing activation of cell growth signaling. These findings are consistent with the thickening of the pulmonary vascular wall in COVID-19 patients. By infecting human pluripotent stem cell-derived cardiomyocytes and SMCs with SARS-CoV-2, Marchiano et al. [184] observed only marginal viral entry in SMCs. On contrary, cardiomyocytes were heavily infected. SARS-CoV-2 infection of cardiomyocytes impaired their electrophysiological and contractile function and frequently led to cardiomyocyte death. These results are in line with the severe cardiac symptoms in COVID-19 patients, particularly suffering from CVD.

In addition, the role of pericytes, precursors of SMCs, is not to be neglected. Pericytes are mural multifunctional cells surrounding ECs of capillaries [189]. They contribute to maintaining hemostatic functions of the endothelium, stabilizing small vessels, and regulating the capillary blood flow. As mentioned above, single-cell RNAseq studies demonstrated that unlike healthy ECs, pericytes and SMCs express high levels of ACE2 [42]. Accordingly, immunohistochemical staining of capillaries might confuse ACE2-positive pericytes or SMCs with ECs. These results support again the indirect activation of ECs by SARS-CoV-2 infecting neighbouring cells, platelets, and inflammatory cells.

Currently, the role of pericytes and SMCs in COVID-19 disease is unclear. Khaddaj-Mallat et al. [185] reported that expression of ACE2 in brain vascular pericytes is significantly increased following interaction with S protein. These infected pericytes underwent profound phenotypic changes expressing more contractile and myofibrogenic proteins. In addition, the spike protein induced oxidative stress, similar to the immune reaction activated by NF-κB signaling pathway supporting hypoxia. All these features are associated with pathomorphology in COVID-19 patients. Furthermore, Tzankov at al. [187] reported that SMCs of COVID-19 patients exhibited increased expression and density of various chondroitin sulfates. These conditions might contribute, e.g., to arterial stiffness. Zhang et al. [186] reported that airway smooth muscles can also be infected by SARS-CoV-2. These cells are stimulated by IL-13 and IL-17, affecting their fate and phenotype. Thus, airway smooth muscles and pro-inflammatory factors may play an important role in the progression and severity of COVID-19 course.

Finally, it remains an open question whether infection of ECs by SARS-CoV-2 might also change the expression profile of SMCs or facilitate their trans-differentiation, a process, which is tuned by interactions between ECs and SMCs [169]. Thus, whether SMCs can be directly infected by SARS-CoV-2 or are indirectly affected by EC signaling and inflammatory response following COVID-19 is yet to be elucidated.

## The role of long noncoding RNAs in viral infection

Cell-to-cell variability of virus infection has been a major unexplained feature in infectious diseases [190]. In recent years, increasing evidence has demonstrated a crucial role of particularly long noncoding RNAs (lncRNAs, >200 nt) in various biological processes, such as epigenetic modification, regulation of transcriptional as well as post-transcriptional gene expression, and aging [58–60,191–193]. Specific interactions of lncRNAs with DNA, mRNA and proteins can modulate chromatin function, mRNA stability and translation as well as cell signaling. LncRNAs also contribute to the severity of CVD [59,60,194–196]. Multiple studies have shown that lncRNA significantly influence vascular permeability, proliferation, migration, phenotypic switching, and apoptosis in the context of vascular dysfunction well as crosstalk between ECs and SMCs [58,61,62,196–198].

LncRNAs may also be of importance in the regulatory network controlling virus entry, innate immunity and infection, as shown for HIV, hepatitis B virus, influenza virus or hepatitis C virus, for example [64,65,199,200]. In particular, lncRNAs have been observed to regulate the expression of interferon (IFN) stimulated genes (ISGs), targeting the corresponding upstream transcription factors and acting through epigenetic modifications [64,200]. For instance, IFN-α induces the expression of lncRNAs lncBST2 and lncISG15, which are positive regulators of the IFN-stimulated genes [201]. On the contrary, e.g. lncRNA NRAV has been found as a negative regulator of antiviral response, promoting the replication of the influenza virus [67].

Regarding their potential protective antiviral function, lncRNAs can bind to the complementary sequences of viral RNA, forming a double-stranded RNA, which is then degraded by the internal RNA-induced silencing complex (RISC) [202]. In general, lncRNA/viral RNA interactions can either repress the viral mRNA translation and inhibit viral replication, or stabilize the viral RNA, enhancing the viral replication [203–205].

A variety of human viruses, such as herpes simplex virus (HSV) [206,207], human immunodeficiency virus (HIV) [206], hepatitis B virus (HBV) [207,208], and SARS-CoV [201] have been shown to dysregulate the expression of several host lncRNAs, increasing infectivity. Among them, the lncRNA HULC (highly up-regulated in liver cancer) and HEIH (highly expressed in HCC) have been reported to play important roles in HBV-related carcinogenesis. Furthermore, HIV infection has been shown to up-regulate the lncRNA nuclear paraspeckle assembly transcript 1 (NEAT1) expression [206]. NEAT1 is essential for the integrity of the nuclear paraspeckle substructure, which is an important subcellular organelle for HIV-1 replication. In addition, the lncRNA called metastasis-associated lung adenocarcinoma transcript 1 (MALAT1) has been up-regulated in HIV-infected T-cells and macrophages, affecting apoptosis of these cells [210,211].

Regarding the innate immune system in the host antiviral response, lncRNA#32 was observed to regulate the expression of ISGs and IFN signaling [63]. Repression of lncRNA #32 significantly increased susceptibility to encephalomyocarditis virus and HBV. In particular, the expression of several chemokines and innate immune response genes was significantly repressed in the lncRNA #32 depleted cells. Other studies demonstrated that the lncRNA negative regulator of antiviral response (NRAV) was highly expressed in cells infected with influenza A virus (IAV) [67]. Furthermore, following infection with HIV or hantavirus, lncRNA NEAT1 interaction with other proteins and transcription factors has been found to repress the IFN-associated genes, facilitating infections [212,213]. Here is to note that many of these lncRNAs, such as NEAT1, MALAT1, and H19 play an important role also in CVD and atherosclerosis [58–60].

It is also worth mentioning that RNA viruses manipulate and hijack the host lncRNAs to promote their replication [199]. For instance, lncRNA–ACOD1 reduces antiviral innate response and is induced by various animal viruses, including Sendai virus, a severe pathogen of mice, and vesicular stomatitis virus infecting equidae, sheep and goat. Following infection, the viral machinery may not only alter the expression pattern to augment the cell conditions for virus replication and dissemination but also avoid and down-regulate the immune response [203,214].

## The role of long noncoding RNAs in vascular dysfunction of COVID-19

As described above, virus infection of host cells changes not only the expression pattern of many lncRNAs but also plays an essential role in antiviral immunity. Thus, the susceptibility to SARS-CoV-2 infection might be defined not only by the surface proteins such as ACE2 and TMPRSS2 but also by the expression of host-derived lncRNAs [215]. In the context of SARS-CoV, infection of various mouse strains (PWK/PhJ (PWK), CAST/EiJ (CAST), 129S1/SvImJ (129/S1), and WSB/EiJ (WSB) mice) appears to be associated with differential expression of several hundred lncRNAs [208]. Most changes correlated with the activation of the IFN response, akin to broad IFN-dependent and independent anti-viral effects of short blunt end double-stranded RNAs [216]. One of these lncRNAs involved in antiviral response was NeST, and it was associated with altered histone methylation of the IFNG promoter, and stimulating IFN-γ gene expression [217]. These data suggest that lncRNAs may particularly affect antiviral responses through the transcriptional activation of IFN-associated genes by recruiting transcription and epigenetic factors.

Emerging clinical evidence suggest that lncRNAs play a decisive role in COVID-19 patients [218–226]. These articles mainly focused on the analysis of lncRNAs as circulating biomarkers in blood using existing genomic databases [219,222–224]. For instance, Li et al. performed whole transcriptome analysis of peripheral blood samples and identified 23 differentially expressed miRNAs and 410 differentially expressed lncRNAs in the COVID-19 samples compared with healthy controls [223]. MALAT1, NEAT1, HOTAIRM1 (HOX transcript antisense RNA M1), VDR (vitamin D receptor), and SNHG6 (small nucleolar RNA host gene 6) were among the identified lncRNAs, and they were confirmed by other studies [209,218,220,221]. These bioinformatic analyses of pre-existing genomic data revealed dysregulation of miRNA/lncRNA interactions and their effects on ACE2 and TMPRSS2 [219], indicating that some lncRNAs might contribute to the regulation of the expression of these infection regulators at the cell surface and thus has an influence on SARS-CoV-2 susceptibility.

Regarding EC dysfunction in COVID-19 patients, short non-coding RNA miR-200c regulating the expression of ACE2 was reported [224]. This goes along with reports at patient levels showing that lncRNA H19 can bind to this family of miRNAs, reducing their availability in the host cell, leading to higher responsiveness to SARS-CoV-2 [226]. To identify cellular pathways during SARS-CoV-2 infection, another study analyzed transcriptome data from human bronchial epithelial cells (NHBE) infected with SARS-CoV-2 [225]. Activating the innate immune response has been shown to up-regulate IFN-responsive genes in particular. Interestingly, changes in the expression of MALAT1 and NEAT1 have also been observed [225]. However, in the context of COVID-19 and the dysfunction of ECs and SMCs, no studies on lncRNAs have been reported so far.

## COVID-19 and the role of endothelial infection in CVD

COVID-19 patients suffering from CVD have a more than 5-fold increased risk of mortality [37]. These individuals are having atherosclerotic plaques and thus are more vulnerable to plaque activation and risk of cardiovascular failure [16,22]. Furthermore, current evidence suggest that COVID-19 is an endothelial disease and that EC dysfunction triggers various associated factors causing excessive inflammation, oxidative stress, coagulopathy, platelet and fibrinogen activation, and thromboembolism [13,95,130,227–230]. Current data indicate that endothelial dysfunction is not limited to the lungs but can be considered a whole-body disease. Approximately 5% of COVID-19 patients develop multiple organ failure and excessive immune response [72,74,158]. Various studies have shown that COVID-19 patients have more often not only pulmonary embolism but also venous thromboembolism with up to 70% [37,95,228,229]. Other associated complications have been observed as well: acute kidney injury, coagulation disorders, and electrolyte disturbances [37,75,128]. Interestingly, most of the cardiac injuries in COVID-19 patients occurred in the absence of coronary thrombosis and were associated with myocarditis, cardiomyopathy, arrhythmias, and inflammatory cytokine dysregulation [37,96]. In addition, it has been reported that COVID-19 patients can develop new cardiovascular events, such as right and left diastolic dysfunction, indicating the involvement of small coronary capillaries [11,231]. The observed diastolic dysfunction was also attributed to nonischemic myocarditis [39,232]. Even if the cardiac complications are associated with the various stages of EC dysfunction, the underlying mechanisms are not sufficiently understood.

Furthermore, SARS-CoV-2 infection may accelerate atherosclerotic plaque formation toward a vulnerable state and vascular thrombosis. Hypertension, obesity, and diabetes mellitus, frequently accompanying patients with CVD, have already been considered risk factors for severe COVID-19 course [12,24,233]. A recent meta-analysis with 125,446 patients found that the most common comorbidity was hypertension (32%), followed by obesity (25%), diabetes (18%), CVD (16%), lung disease (9%), and chronic kidney disease (6%) [15,135]. Additional risk factor associated with the death of patients with COVID-19 is older age [234]. The comorbidity in the elderly significantly correlates with a high level of D-dimer. More than 20% of COVID-19 patients with severe course have higher blood pressure (HBP) rates exceeding 50% [23]. The contribution of hypertension to the morbidity and mortality in COVID-19 is however still unclear. High blood pressure (>50%) affects various components of cell-mediated immunity that are normally protective against viral infections [23]. Furthermore, hypertension stimulates overexpression of various cytokines, particularly interferon (IFN)-γ and TNF-α [32,74,158]. These cytokines affect renal epithelium and raise blood pressure by increasing oxidative stress and renal sodium reabsorption [23]. Plasma levels of IL-6 are also associated with hypertension as well as higher morbidity of COVID-19 patients [235]. Increased blood pressure predisposes also endothelial injury. Further accompanying diseases of COVID-19 are frequently endotheliitis and vasculitis, however, not only in the pulmonary tract but also in other organs such as kidney [26,31], liver ]31], intestine [127], or heart [128]. In addition, vascular occlusion, stiffness, and altered reactivity have been reported [26,73,236].

Interestingly, venous complications in COVID-19 patients have been more often described than e.g. arterial complications such as on the peripheral arterial system [37,95,229]. Even if it seems that only less than 1% of COVID-19 patients develop systemic arterial thrombotic events, the mortality is high with up to 30% [37]. Thromboembolism in peripheral vessels is less predictable in patients infected by SARS-CoV-2 than in the usual atherosclerotic patients suffering from peripheral arterial disease (PAD). In addition, COVID-19 patients experience more frequently ischemic stroke, with an incidence of 0.05–5.7% [21,37,95–97].

The dysregulation of the normal functions of ECs in COVID-19 patients contributes also to the thrombo-inflammatory storm, formation of blood clot, diffuse coagulopathy, and multiple organ failure, which are frequently associated with mortality and severe course of the disease [36]. These pathologies affect not only the lung capillaries but also to other blood vessels. COVID-19 disease has been described as being accompanied by arterial and venous thrombotic events, particularly in patients suffering from CVD [95,116,237]. Up to 30% of COVID-19 patients admitted to the intensive care unit acquired various thrombotic complications [74,131,237]. The pathophysiology of coagulopathy associated with COVID-19 disease involves several pathways. The initial trigger is mainly the systemic inflammatory response following SARS-CoV-2 infection, activating various coagulation pathways. Accordingly, COVID-19 patients showed increased fibrinogen and D-dimer levels [116,227]. Interestingly however, only a few patients with COVID-19 developed disseminated intravascular coagulation, compared with patients with septic shock (30–40%). The endothelial damage can be further facilitated by very high levels of von Willebrand factor (VWF) in the blood circulation, which in turn contributes to the high inflammatory reaction following SARS-CoV-2 infection [238]. Furthermore, the severe course of COVID-19 leads to hypoxemic pneumonia and hypoxemia in the pulmonary capillaries, resulting in vasoconstriction. Vessel narrowing reduces in turn blood flow in the arteries, thereby facilitating vascular occlusion [116]. In addition, vascular thrombosis and vasculitis have been frequently described as common post mortem pathological findings in COVID-19 patients [14,32].

In summary, the global dysfunction of ECs together with the total or partial collapse of the endothelial barrier represents the critical step in the development of the severe course of COVID-19 disease, leading to multiorgan failure and sepsis [116]. The underlying pathomechanism is the endothelial dysfunction and activation of various inflammatory and adhesive molecules in or through changes in the expression pattern of ECs. The detachment of SARS-CoV-2 infected ECs may disseminate the virus through the circulation and reach other distant organs. These cells can in turn contribute to various thromboembolic events. Nevertheless, it is still unclear how vascular ECs are targeted by SARS-CoV-2. Moreover, it is unknown, how SARS-CoV-2 contributes to vascular complications and where is the link between cardiovascular risk factors such as ageing and accompanying diseases and COVID-19 pathologies.

## Conclusion

Although COVID-19 is primarily a respiratory disease, it has a significant impact on the cardiovascular system, and vice versa, a dysregulated cardiovascular system appears to exacerbate COVID-19. Particularly COVID-19 patients with CVD frequently develop more severe and complicated disease course than patients without CVD. A common denominator of CVD is the dysfunction of vascular cells, increased permeability, endothelial-to-mesenchymal transition, inflammation, and coagulation. It has been assumed that clinical complications are due to SARS-CoV-2 infection of ECs through the ACE2 receptor pathway. However, recent studies have shown significant discrepancies and controversial results, suggesting that noncanonical viral interference or immune reactions with ECs may account for EC dysfunction. Such processes might comprise alternative cell entry pathways of SARS-CoV-2, e.g. involving ADAM17, HSPG, sialic acid, NRP or CD209L, although further studies are required to support such assumption. Regardless, vascular dysfunction in CVD patients might facilitate and accelerate viral entry and infection of damaged ECs and also SMCs. Consequently, it is important to explore the infection mechanism in vascular cells in the context of cardiovascular disorders. Likewise, the role of SMCs in COVID-19 merits further investigations. Other promising areas are diagnostic and therapeutic approaches against COVID-19, in particular considering lncRNAs in the context of CVD. Notably, the importance of lncRNAs is increasingly appreciated, also due to advances in RNAseq technology and single cell analyses addressing mechanisms of infections by considering the ubiquitous nature of cell-to-cell variability [190]. Such approaches might further illuminate the role of lncRNAs in SARS-CoV-2 infection and open new opportunities for drug and therapeutic strategies, particularly in the context of CVD.

## Data Availability

No datasets have been used in producing this work.

## Abbreviations

ACE2: angiotensin-converting enzyme 2
ADAM17: disintegrin and metalloproteinase domain-containing protein 17
APN: alanine aminopeptidase
AT1R: AT1 receptor
AT2R: AT2 receptor
COPD: chronic obstructive pulmonary disease
CoV: coronavirus
COVID-19: coronavirus disease 2019
CRP: C-reactive protein
CVD: cardiovascular disorder
DMV: double-membrane vesicle
DPP4: dipeptidyl peptidase-4
EC: endothelial cell
EGFR: epidermal growth factor receptor
ENaC: epithelial sodium channel-α
FGF-2: fibroblast growth factor-2
HBV: human hepatitis B virus
HCAEC: human coronary artery endothelial cell
HIV: human immunodeficiency virus
HLMVEC: human lung microvascular endothelial cell
HPAEC: human pulmonary arterial cell
HSPG: heparan sulphate proteoglycans
HUVEC: human umbilical vein endothelial cell
IAV: influenza A virus
ICAM-1: intercellular adhesion molecule 1
IL: interleukin
INF: interferon
lncRNA: long noncoding RNA
MALAT1: metastasis-associated lung adenocarcinoma transcript 1
MasR: MAS receptor
MCP-1: monocyte chemoattractant protein-1
NEAT1: nuclear assembly transcript 1
NET: neutrophil extracellular trap
NRP1: neuropilin-1
ROS: reactive oxygen species
SAA: serum amyloid A
SARS: severe acute respiratory syndrome
SMC: smooth muscle cell
TMPRSS2: transmembrane protease serine subtype 2
TNF-α: tumor necrosis factor α
VCAM-1: vascular cell adhesion molecule 1
VEGF: vascular endothelial growth factor
